# Compare the effect of noninvasive ventilation and tracheotomy in critically ill mechanically ventilated neurosurgical patients: a retrospective observe cohort study

**DOI:** 10.1186/s12883-019-1297-3

**Published:** 2019-05-01

**Authors:** Meiling Dong, Yongfang Zhou, Jing Yang, Jie Yang, Xuelian Liao, Yan Kang

**Affiliations:** Department of critical care medicine, West China Hospital of Sichuan University, Sichuan Province, Chengdu, 610041 China

**Keywords:** Noninvasive ventilation, Brain injury, Tracheotomy, Postoperative pulmonary infection

## Abstract

**Objective:**

Patients with severe brain injury is usual at high risk of extubation failure, despite of those with no/minor primary respiratory problem, majority of them still needs long term respiratory support and has severe pulmonary complications. This retrospective study aimed to compare the effect of noninvasive ventilation (NIV) and tracheotomy on the prognosis in critically ill mechanically ventilated neurosurgical patients.

**Methods:**

This is a single center, retrospective observe cohort study. Postoperative patients with brain injury consecutively admitted to ICU from November 1st, 2015 through February 28th, 2017, who had received invasive mechanical ventilation more than 48 h were screened, those who received NIV or tracheotomy procedure, meanwhile with Glasgow Coma Scale (GCS) score between 8 and 13 points before using NIV or undergoing tracheotomy, were retrospectively included in this study. The demographic data and clinical main outcomes such as ICU and hospital mortality, time of mechanical ventilation, length of ICU and hospital were collected. The primary outcome was the incidence of postoperative pulmonary infection between two groups.

**Results:**

77 patients were included in this study. 33 patients received NIV, and 44 patients received tracheotomy through the ICU duration. The incidence of postoperative pulmonary infection in NIV group was significantly lower than that in tracheotomy group (54.5% VS 84.1%, *P* < 0.05), Application of NIV was associated with shorter duration of invasive mechanical ventilation ([median 123.0 h VS 195.0 h, *P* < 0.05). Moreover, GCS score at ICU discharge, as well as the difference of GCS score between at admission to ICU and ICU discharge were also better than the tracheotomy group (*P* < 0.001).

**Conclusion:**

Compared with tracheotomy, use of NIV after extubation in critically ill mechanically ventilated neurosurgical patients may be associated with lower incidence of postoperative pulmonary infection, shorter duration of invasive mechanical ventilation and better improvement in brain function. Further studies need to verify the effect of NIV in this kind of patients.

## Introduction

Brain injury is a growing problem of public health and social economy in the world. In United States, about 235,000 patients are admitted to hospital every year because of traumatic brain injury, and the annual death toll is about 50,000. The total cost of hospitalization due to traumatic brain injury in 2010 amounted $21 billion to 400 million [1, 2]. In China, the incidence of traumatic brain injury in the age of over 65 years has increased by 7.78% annually, with an average hospitalization cost of $795 and a fatality rate of 9.38% [3]. In order to maintain respiration, optimize oxygenation and protect airway, patients with brain injury usually need Mechanical Ventilation (MV) in ICU. The vast majority of brain injury patients without respiratory problem, when the patient acute physiological disorder has improved, most patients can wean from MV as soon as possible. However, more than 20% of patients still need ventilator support after 21 days [4]. These patients may need tracheotomy or noninvasive ventilation (NIV) for further treatment. Previous studies have shown that long-term mechanical ventilation were associated with a lot of pulmonary complications in patients with severe brain injury, such as ventilator-associated lung injury, pulmonary infection, diaphragm dysfunction, pulmonary embolism and so on, and increased the mortality of disease [5]. Relevant studies also demonstrated that, in brain-injured patients,the incidence of pulmonary infection was 20%, the rate of reintubation caused by extubation failure was from 10 to 15%, and the incidence of pneumonia after extubation failure was as high as 85% [6–8].

NIV has become a common alternative to invasive ventilation at present, several studies have shown that NIV weaning strategy was superior to invasive weaning strategy in hypercapnic respiratory failure patients, and it could significantly reduce the re-intubation rate, the incidence of ventilator associated pneumonia (VAP), duration of mechanical ventilation, and the ICU and hospital length of stay [9–12]. But there were few studies and literatures demonstrated the benefits of NIV in acute hypoxemic respiratory failure, especially in the brain injury patients. Lack of research has caused considerable confusion to clinical workers: could NIV improve the prognosis of patients who accepted brain injury surgery? Therefore, we designed this retrospective study to explore the impact of NIV on the prognosis of patients who accepted brain injury surgery compared with tracheotomy.

## Materials and methods

### Patients

This was a retrospective, observe cohort study of data from patients after brain injury surgery at the intensive care unit (ICU) of West China Hospital, Sichuan University, Chengdu, Sichuan province, China, between November 1st, 2015 and February 28th, 2017. The ethics committee of West China Hospital approved this study. Critically ill mechanically ventilated neurosurgical patients admitted to our ICU will be managed according to different disease protocols, these protocols are based on guidelines and the clinical experience of physicians. Patients older than 18 and less than 85, who still needed invasive mechanical ventilation longer than 48 h after neurosurgery, were recruited in our study. Patients were excluded if they were pregnant, without NIV and tracheotomy treatment during total ICU stay, with Glasgow Coma Scale (GCS) score before NIV or tracheotomy less than 8 and more than 12 points, and with multiple organ failure, hemodynamic instability and tracheostomy prior to ICU admission.

### Methods

According to inclusion and exclusion criteria, the demographic data and clinical main outcomes of selected patients were collected. The data included demographic characteristics of enrolled patients, information about perioperative period, and other basic information related to respiratory system and nervous system from ICU admission to ICU discharge or maximum of 28 days. Of which, the respiratory system data included daily blood gas, daily ventilator parameters, daily major respiratory events (such as extubation, wearing NIV, tracheotomy, changing cannula, etc.), daily pulmonary complications, daily lung imaging, daily clinical pulmonary infection score (CPIS) and so on. The nervous system data included the occurrence of cranial complications, intracranial pressure, daily GCS score and so on. According to whether NIV or tracheotomy was performed after invasive mechanical ventilation during ICU stay, the patients were divided into NIV group and tracheotomy group.

The primary outcome in this study was the incidence of postoperative pulmonary infection in patient ICU stay. Simplified version of the clinical pulmonary infection score (sCPIS) was used to evaluate the pulmonary infection of enrolled patients [13]. The sCPIS contains five variables and the calculation is displayed on Table 1. If the total points is greater than or equal to 6 points, we considered a postoperative pulmonary infection [14, 15]. The secondary outcomes were the incidence of other pulmonary complications, re-intubation rate, duration of mechanical ventilation, and the length of ICU stay and hospital stay.

**Table 1:** Simplified version of the clinical pulmonary infection score (sCPIS)

Variables	Value	Points
Chest radiograph	No infiltrate	0
	Patchy or diffuse infiltrate	1
	Localized infiltrate	2
Tracheal secretions	Few	0
	Moderate	1
	Large	2
	Purulent	+ 1
Temperature °C	≥36.5 and ≤ 38.4	0
	≥38.5 and ≤ 38.9	1
	≥39.0 or ≤ 36.0	2
Oxygenation PaO_2_/FIO_2_, mmHg	> 240 or presence of ARDS	0
	≤240 and absence of ARDS	2
Blood leukocytes per mm^3^	≥4000 and ≤ 11,000	0
	< 4000 or > 11,000	1

*ARDS* acute respiratory distress syndrome. Total points for sCPIS varied from 1 to 10 points

### Statistical analysis

SPSS22.0 was used for statistical analysis of data in our study. Continuous variables with a normal distribution were expressed as the mean ± standard deviation, and inter-group comparison was analyzed by the Student’s t test. Continuous variables with non-normal distribution were expressed as the median and interquartile ranges (IQR), and their differences were compared with the Mann-Whitney U test. Dichotomous or nominal categorical variables were expressed as percentages, and analyzed by either the Pearson Chi-square or Fisher’s exact test. A two-sided *P* value of < 0.05 was considered to statistical significance.

## Results

A total of 1278 patients admitted to SICU and NICU in our hospital from November 2015 to February 2017 were screened. 77 patients were enrolled in this study finally, including 33 patients who had only used NIV (NIV group) and 44 patients who had only undergone tracheotomy (tracheotomy group) after invasive mechanical ventilation.

Table 2 showed the patient baseline characteristics between two groups. There was no difference in any baseline characteristics between two groups(*P* > 0.05). The level of consciousness and the criticality of the patient were similar between two groups, with no significant differences of APACHE II score and GCS score before NIV or tracheotomy were observed between two groups.

**Table 2:** Baseline characteristics of patients

Characteristic	NIV(*n* = 33)	Tracheotomy(*n* = 44)	*P* value
Age, years (Median, IQR)	57 (48–64)	61.5 (50.5–66.7)	0.187
Gender, male, n (%)	15 (45.5%)	22 (50.0%)	0.693
Smoke, n (%)	9 (27.3%)	10 (22.7%)	0.647
Body Mass Index (mean ± SD)	23.74 ± 3.53	23.17 ± 2.85	0.434
Basic disease, n (%)			
COPD	4 (12.1%)	3 (6.8%)	0.454
Hypertension	17 (51.5%)	22 (50.0%)	0.895
Cerebrovascular events	7 (21.2%)	6 (13.6%)	0.38
Basic condition before operation, n (%)			
Vomit	12 (36.4%)	13 (29.5%)	0.527
Aspiration	4 (12.1%)	8 (18.2%)	0.468
Epilepsy	0 (0.0%)	2 (4.5%)	0.504
pulmonary infection	13 (39.4%)	10 (22.7%)	0.114
coma before operation, n (%)	10 (30.3%)	19 (43.2%)	0.248
Emergency operation, n (%)	16 (48.5%)	23 (52.3%)	0.644
APACHE II score at ICU admission (Median, IQR)	17.0 (15.5–20.0)	17.5 (16.0–20.0)	0.623
GCS score at ICU admission (Median, IQR)	9.0 (8.0–9.5)	9.0 (8.0–9.0)	0.107
Coma after operation, n (%)	28 (84.8%)	46 (76.7%)	0.349
GCS score before NIV or tracheotomy (Median, IQR)	9.0 (9.0–10.5)	8.0 (8.0–10.0)	0.115
ICU duration before NIV or tracheotomy, days (Median, IQR)	5.0 (3.0–7.5)	4.0 (3.0–7.0)	0.104
IMV time before NIV or tracheotomy, hours (Median, IQR)	115 (61.0–178.5)	100 (63.13–164.5)	0.607

Data are expressed as the mean ± standard deviation, and as the median and interquartile ranges (IQR), or percentages. *SD* standard deviation, *COPD* Chronic Obstructive Pulmonary Disease, *APACHE II* Acute Physiology and Chronic Health Evaluation; *ICU* intensive care unit, *GCS* Glasgow Coma Scale, *IMV* Invasive mechanical ventilation

**Fig. 1:**
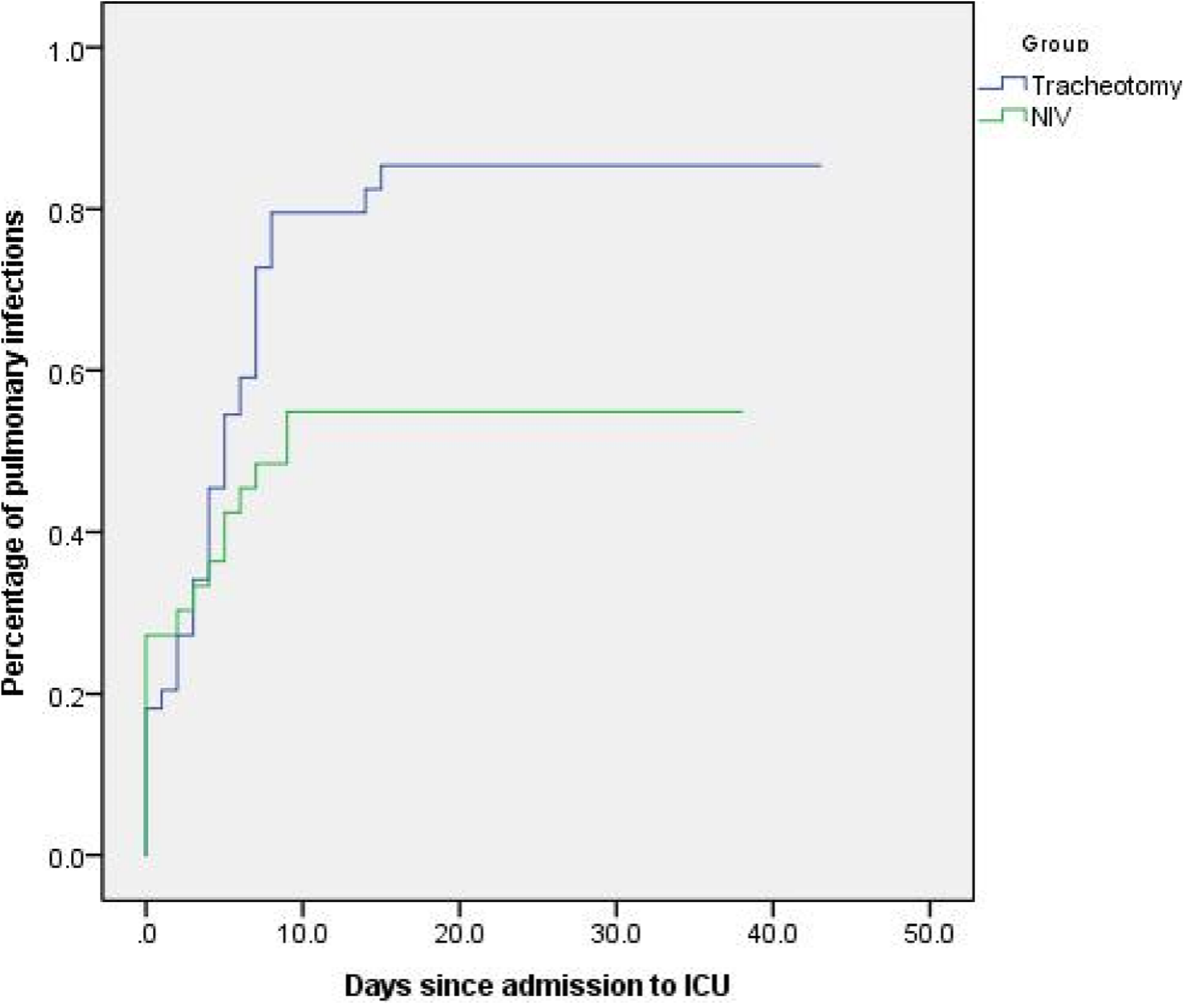
Percentage of pulmonary infections since admission to ICU in the NIV and tracheotomy groups. *P* < 0.05 by log-rank test

Study outcomes are showed in Table 3. The incidence of postoperative pulmonary infection in NIV group was lower than tracheotomy group [54.5% vs.84.1%, *P* < 0.05] (Table 3 and Fig. 1), and the duration of invasive mechanical ventilation was shorter than tracheotomy group(*P* < 0.05). In addition, the length of ICU and hospital stay in the NIV group were also shorter than tracheotomy group. And we found that NIV group showed a higher GCS score at discharge ICU and GCS score difference between admission and discharge ICU. This suggested that NIV may accelerate the recovery of consciousness in brain injury surgery patients.

**Table 3:** Primary and secondary outcomes of patients

Variable	NIV(n = 33)	Tracheotomy(n = 44)	*P* value
Postoperative pulmonary infection, n(%)	18 (54.5%)	37 (84.1%)	0.005^a^
Atelectasis, n (%)	2 (6.1%)	5 (11.4%)	0.692
Mortality in ICU, n(%)	1 (3.0%)	6 (8.0%)	0.228
GCS score at ICU discharge	12.0 (13.0–14.0)	10.0 (8.0–12.0)	< 0.001^b^
△GCS score at ICU	4.0 (3.0–5.0)	0.5 (0.0–2.0)	< 0.001^b^
Total mechanical ventilation time, hours (Median, IQR)	218.0 (142.0–294.5)	195.0 (127.3–372.3)	0.658
Invasive mechanical ventilation time, hours (Median, IQR)	123.0 (89.5–218.0)	195.0 (127.3–372.3)	0.005
ICU duration, days (Median, IQR)	14.0 (9.0–22.5)	18.0 (13.3–26.8)	0.059
Hospital duration, days (Median, IQR)	22.0 (17.0–29.5)	28.5 (18.25–41.75)	0.151

Data are expressed as the median and interquartile ranges (IQR), or percentages. *COPD* Chronic Obstructive Pulmonary Disease, *APACHE II* Acute Physiology and Chronic Health Evaluation; *ICU* intensive care unit, *GCS* Glasgow Coma Scale, △GCS score at ICU: GCS score difference between admission and discharge ICU; a: *P* < 0.05; b: *P* < 0.001

## Discussion

Our study demonstrated that NIV was associated with lower postoperative pulmonary infection incidence, shorter duration of invasive mechanical ventilation, better level of consciousness at ICU discharge, compared with tracheotomy.

When patients do not meet the MV withdrawal criteria, some medical workers may choose tracheotomy and some may choose early NIV intervention. There still are some controversies about the possible benefits of NIV compared with tracheotomy. The strength of mechanical ventilation support is generally low in patients with brain injury, however, due to disturbance of consciousness or damage of brain stem, airway protective reflexes such as cough and swallow weakened or disappeared, and artificial airway continue to be needed to maintain airway unobstructed [16]. But for patients without other mechanical ventilation indications, people who need respiratory support and artificial airway may be different. For patients who only need respiratory support, the complications caused by artificial airway cannot be ignored [17–19]. Early and timely application of NIV can reduce complications of artificial airway, avoid delayed extubation and related complications, and shorten length of ICU and hospital stay, leading to improve the prognosis of patients [9–11, 17]. Coplin et al. found that 48% (29/60) of patients with GCS score < 8 had delayed extubation compared with 10.5% (8/76) of patients with GCS > 8 (*P* < 0.05) [20]. Particularly interesting was the fact that there was almost no difference in consciousness on the day when the extubation criteria were met and the day of extubation in the delayed extubation patients. At the same time, the study also found that patients with swallow reflex and cough reflex disappeared or weakened could also extubated successfully. These findings challenge the conventional indicators of airway protection (such as swallow reflex, cough reflex, GCS score). These indicators which are not only used to evaluate airway protection ability but also commonly used by clinicians to assess the failure rate of noninvasive ventilation [21, 22]. Therefore, so are these indicators still important for patients with brain injury? In this study, the median GCS score of NIV patients was 9 points, which was lower than the general criteria (GCS > 13). In 33 patients with noninvasive ventilation, only 4 patients failed in noninvasive ventilation, and 3 cases of them used the noninvasive ventilation for respiratory failure after extubation, for the preventive treatment of respiratory failure, noninvasive ventilation obviously has a good effect in this study [23].

Compared with NIV, tracheotomy is an invasive operation and also very common in ICU. At present, more and more ICU doctors can perform percutaneous tracheotomy beside the bed. Tracheotomy has many potential advantages, and it reduces the risk of self-extubate, airway injury, respiratory resistance, facilitating drainage of secretions, better tolerance, and less sedation and analgesia requirement [24]. McIntyre et al. first suggested that patients who need artificial airway more than 21 days should be considered tracheotomy [25]. And then, D’Amelio et al. found that early tracheotomy in patients with brain injury (mechanical ventilation days less than 7 days) can reduce the duration of mechanical ventilation, length of ICU and hospital stay [26]. Subsequently, a large RCT study demonstrated that early tracheotomy (6–8 days after mechanical ventilation) did not significantly reduce the incidence of ventilator-associated pneumonia, 28-day and one-year mortality, and time of hospital stay compared with later tracheotomy (13–15 days after mechanical ventilation) [27]. Therefore, there is no accepted conclusion about the appropriate timing and indication of tracheotomy in patients with brain injury, but the only fact which is generally accepted is that when the GCS score is still less than 8 points after 7 days of mechanical ventilation, early tracheotomy is recommended for patients pre estimate longer mechanical ventilation [28]. All of the patient enrolled in our study chose to undergo tracheotomy or NIV only after their craniocerebral state was stable, our ICU doctor will evaluate the patient’s condition every day. If the patient’s condition is stable, we will stop using sedation and discuss whether tracheotomy or NIV was undergone, so the GCS scores of the patients with tracheotomy in this study were all higher than or equal to 8 points, and 78% (47/60) of the patients underwent early tracheotomy (mechanical ventilation days < 7). This may be the reason why the incidence of pulmonary infection, duration of invasive mechanical ventilation, time of stay in ICU and hospital in the tracheotomy group were higher than those in the NIV group. In addition, the risk factors of postoperative pulmonary infection in brain injury surgery patients included: older age, longer coma time, complicated with chronic underlying diseases, smoking history, tracheal intubation, tracheotomy, tracheotomy intubation for a long time, long hospitalization days, and irrational use of antibiotics and the like.

There are some limitations in this study, firstly, we cannot completely balance the bias caused by potential unknown confounding factors, for example, snoring, lingual drop, tracheal displacement, analgesic and sedative drug use. And most of all, NIV is a respiratory treatment influenced by several factors easily, and staff training is a key one. Medical workers with different clinical experience may have a different understanding of the clinical application of NIV [29]. What’s more, because this study was a retrospective cohort study that was not a prospective randomized controlled trial, the baseline characteristics of the NIV and tracheotomy patients were not well balanced, for instance, although there was no significant statistical difference in GCS scores prior to NIV or tracheotomy between NIV and tracheotomy patients, the NIV group had a trend of a higher GCS score before NIV, which may cause selective bias in the results. Therefore, more comprehensive prospective studys with a larger sample size are necessary to explain these issues better, further explore the application of noninvasive ventilation and tracheotomy in patients with brain injury, and identify the better respiratory support and airway management methods and timing to maximize patient benefits.

## Conclusion

As far as we know, this is the first study investigating the impact of NIV and tracheotomy on the prognosis in critically ill mechanically ventilated neurosurgical patients. Compared with patients underwent tracheotomy, the patients who accepted NIV has a lower incidence of postoperative pulmonary infection, shorter duration of invasive mechanical ventilation, higher GCS score at discharge ICU, as well as significant GCS score difference between ICU admission and discharge. Researches about the impact of noninvasive ventilation and tracheotomy on the prognosis of neurosurgery patients are necessary in the future. Furthermore, the effect of NIV in those patients still requires more efforts.

## Abbreviations

APACHE II: Acute Physiology and Chronic Health Evaluation
ARDS: Acute respiratory distress syndrome
COPD: Chronic Obstructive Pulmonary Disease
CPIS: Ventilator associated pneumonia
GCS: Glasgow Coma Scale
ICU: Intensive Care Unit
IMV: Invasive mechanical ventilation
IQR: Interquartile ranges
MV: Mechanical Ventilation
NIV: Noninvasive ventilation
RCT: Randomized Controlled Trials
sCPIS: Simplified version of the clinical pulmonary infection score
SD: Standard deviation
VAP: Ventilator associated pneumonia
